# Negotiating health: A qualitative analysis of home smoking rules among families with medically vulnerable infants

**DOI:** 10.18332/tid/210927

**Published:** 2026-02-06

**Authors:** Yolanda R. Villarreal, Thomas F. Northrup, Stephen M. Fischer, Jackson S. Norwood, Angela L. Stotts

**Affiliations:** 1 Department of Family and Community Medicine, McGovern Medical School, The University of Texas Health Science Center at Houston, Houston, United States

**Keywords:** secondhand smoke exposure, maternal health, motivational interviewing, collaborative care, smoking cessation

## Abstract

**INTRODUCTION:**

Approximately 5000 child deaths are attributed to secondhand smoke exposure (SHSe) annually, which is three times the number of childhood cancers combined. Infants, medically fragile infants, are highly vulnerable to the harmful effects of SHSe, including respiratory infections and sudden infant death syndrome. While having a home smoking ban may mitigate these risks, implementation remains a challenge for many families. Our primary aim was to explore the familial and sociocultural factors associated with smoking-ban initiation and maintenance in households with medically fragile infants.

**METHODS:**

Qualitative interviews were conducted with 20 mothers participating in a behavioral intervention from 2015 to 2016 aimed at reducing SHSe in infants discharged from a large urban, children’s hospital in Houston, Texas. Interviews explored family structure, cultural influences, social networks, and smoking history. Thematic analysis was used to identify key themes.

**RESULTS:**

Three primary themes emerged: 1) Household structure and power dynamics – mothers in multigenerational homes often lacked authority to enforce smoking bans, especially when the primary authority figure was a smoker; 2) Sole responsibility – mothers felt burdened as the only advocates for SHSe reduction, often without support from other household members; and 3) Variable level of support for SHS bans – while emotional and logistical support was common during infants’ hospital stays, this support rarely extended to smoking-related behavior change. Participants felt these factors significantly influenced smoking-ban initiation and sustainability.

**CONCLUSIONS:**

Findings underscore the need to move beyond individual-level interventions and engage the broader household context. Intervention sessions should include all household members – particularly individuals who smoke – and incorporate collaborative care models that offer behavioral counseling, pharmacological aids (e.g. nicotine replacement therapy), and real-time feedback technologies. Tailoring interventions to reflect household power structures and support systems may enhance their effectiveness in reducing SHSe and protecting medically vulnerable infants.

## INTRODUCTION

Approximately 5000 child deaths each year are due to secondhand smoke exposure (SHSe), which is three times the number of childhood cancers combined^1,2^. Recent research found that children exposed to SHS show significantly higher levels of exposure to volatile organic compounds (VOC) compared to SHS-exposed adults^3^. Infants, particularly those who are medically vulnerable, are especially susceptible to the effects of SHSe (e.g. respiratory infections, SIDS)^4^. Understanding how social relationships and social contingencies impact SHSe in children is an important step in understanding and intervening with families to reduce SHSe within their homes.

Interventions targeting SHSe in the home have ranged from minimal or brief counseling by a physician or nurse to multiple in-home sessions by trained counselors utilizing cognitive and behavioral strategies, yet none has been uniformly effective^5-7^. Historically, SHSe interventions have targeted motivation and behavioral practices of individual(s), i.e. mothers or mothers and partners, with less emphasis on contextual or environmental factors influencing change^8^. Hovell and Hughes^9^, however, put forth a broader Behavioral Ecological Model of SHSe identifying cultural and social contingencies as necessary to fully understand behavior change. In a review by Ferris et al.^10^ several social and environmental factors were identified by parents approached in medical settings as barriers to reducing child smoke exposure, e.g. lack of hospital policy supporting physicians to prioritize SHSe when talking with patients. Further, in recent years, cognitive behavioral therapy models have begun to emphasize the context of behavior change and to incorporate contextual interventions^11-15^.

In most SHSe interventions, a primary behavioral target is the implementation and maintenance of home and/or car smoking bans, which can be challenging for many individuals and families. Family members may hold different values and beliefs about SHSe-reduction importance, strategies, and other related factors, making family and household composition important considerations when developing and implementing a SHSe intervention. In a study on household-smoking-ban initiation for low-income families of children with (n=91) and without (n=91) asthma, families with both parents currently living together, living in a single family home, and having a child with asthma contributed to the successful enforcement of strict smoking bans^16^. Single-parent status was significantly associated with lower odds of living in a smoke-free home in other studies, even when accounting for variables contributing to economic disadvantage^17,18^. Multigenerational households and gender norms may also pose barriers to home-smoking-ban implementation, although little research has been conducted to explore this issue. A review of studies from 2000 to 2008 found that women’s capacity to control their children’s exposure to SHS was impacted by unequal power differences in the home^19^. Understanding external factors impacting a primary caregiver’s ability to affect change in household smoking policies is critical to improving the effects of such interventions.

Infants discharged from a neonatal intensive care unit (NICU) are medically fragile, often born with low birth weight and at higher risk for respiratory illness and disease, and arguably are most vulnerable to SHSe^20,21^. Compared to a single health intervention session focused on SHSe-reduction strategies, our Baby’s Breath II study testing a 4-session motivational interviewing plus financial incentives intervention with mothers of NICU infants failed to demonstrate a difference in infant cotinine levels at follow-up^22^. To inform future intervention development to reduce children’s SHSe, we conducted qualitative interviews to better understand social and contextual factors influencing mothers’ ability to implement smoking bans.

## METHODS

We conducted qualitative interviews with 20 mothers who reported at least one smoker living in the home and had infants discharged from a NICU of a large (1200 admissions/year) children’s hospital. Purposive sampling was undertaken and participants were drawn from mothers (n=360) participating in the Baby’s Breath II (BBII) study [NCT01726062; 2012-2018] which tested a motivational and behavioral intervention to reduce SHSe using a parallel group, randomized, controlled design. Four motivational counseling sessions were conducted: 2 in the hospital and 2 in the home. Sessions targeted protective strategies like smoking outside and home and/or car smoking bans using health information, values identification, goal-setting, and readiness ruler exercises in line with previous motivational interviewing protocols. Financial incentives were delivered for session attendance and cotinine-free infant urine samples post-discharge. Although the overall intervention effect on infant cotinine was not significant, infants of mothers in the intervention group with high baseline readiness and confidence to protect their infant had significantly lower cotinine levels relative to those in the control group^22^.

BBII study completers between 2015 and 2016 were invited to participate in a 60-minute qualitative interview and received $30 incentive for their participation. Interviews focused on respondents’: 1) experience with smoking/SHSe; 2) description of the impact of smoking/SHSe on everyday life; and 3) perceptions of the effect of smoking/SHSe on family relationships. Interviews were audio recorded to ensure accuracy and were conducted in participants’ homes where privacy and minimal interruptions were ensured. An empirically-guided, semi-structured interview guide was developed, consisting of open-ended questions and supplemented by probing questions to elicit a richer set of responses for each topic. In an effort to ensure consistency across interviews, and mitigate any potential interviewer bias, a standardized interview protocol was developed. Additionally, before interviews occurred, the interviewer was trained on active listening techniques and neutral questioning.

Data were analyzed using a thematic analysis approach, which involved systematically identifying, organizing, and interpreting patterns of meaning across the dataset^23,24^. Using content analysis methodology, transcripts were reviewed iteratively by the research team to develop a preliminary coding framework grounded in both the interview guide and emergent themes^25^. The coding framework was entered into NVIVO 14; and three investigators coded each interview, applying the coding framework. Codes were grouped into broader categories, and key themes were refined through team discussions to ensure consistency and depth of interpretation. This method allowed for a nuanced understanding of the social and contextual factors influencing household smoking bans. Coding discrepancies, while minimal, were resolved via reoccurring discussion by the coding team and review of raw data^26^. High inter-coder agreement was reached (κ >0.93)^26^. Coder bias was addressed via a reflexive approach^27^, consisting of regularly reflecting on potential biases and their influence on data interpretations.

## RESULTS

Participants had a mean age of 27 years (SD=5.9), and tended to be Black (55%) or Hispanic (40%), and identified as non-smokers (90%). The majority of women were unemployed (80%), received Medicaid (85%) and had completed on average of 11.3 years (SD=2.8) of school. Forty percent lived in a home with an annual household income under $15000, and with a parent or extended family member. Attendance at intervention sessions by other household members varied by session (Table 1). On average, participants had been pregnant nearly 3 times (mean=2.9, SD=1.8) and had given birth twice before (mean=2.2, SD=1.6).

**Table 1:** Characteristics of mothers with medically vulnerable infants (N=20)

*Characteristics*	*n (%)*
**Race/ethnicity**	
Black/African American	11 (55)
Hispanic White	8 (40)
Non-Hispanic White	1 (5)
**Household status**	
Living with partner	10 (50)
Living with parents/extended family	8 (40)
Living alone	2 (10)
**Work status**	
Employed	4 (20)
Unemployed	16 (80)
**Household income** ($)	
<15000	8 (40)
15000–24999	6 (30)
35000–44999	1 (5)
≥55000	2 (10)
**Medicaid status^a^**	
Yes	17 (85)
No	3 (15)
**Smoking status^b^**	
No smoking	18 (90)
Smoking	2 (10)
**Planned pregnancy**	
Yes	4 (20)
No	16 (80)
**Others present at session^c^**	
Session 1	4 (29)
Session 2	7 (50)
Session 3	6 (43)
Session 4	6 (43)
***Measure***	***Mean (SD)***
Age (years)	27.3 (5.9)
Education level (years)	11.3 (2.8)
Number of pregnancies	2.9 (1.8)
Number of births	2.2 (1.6)

Percentages may not total 100 due to rounding.

a Refers to enrollment at the time of the infant’s hospitalization.

b Reflects self-reported behavior during the study period.

c Indicates the number of participants who had additional individuals (e.g. family members, partners) present during each interview session. Participants were graduates of the Babies Breath II Study [NCT0172606; 2012-2018] with qualitative data collection in 2015–2016.

Participants identified three main themes that influenced their ability to implement and maintain smoking bans: 1) Household structure and power dynamics – living in a multigenerational home (i.e. with extended family members) versus single-generational home (i.e. alone or with a partner) impacts their ability to implement a smoking ban; 2) Sole responsibility – mothers as the one responsible for conveying intervention materials and protecting their infants from SHSe, often without support from partners or family members; and 3) Variable level of support for SHS bans – support from family members and friends was common at times but did not extend to smoking-related behavior change (Table 2).

**Table 2:** Themes influencing home smoking ban implementation among mothers of medically vulnerable infants

*Theme*	*Description*
**Household structure and power dynamics**	Participants in multigenerational homes lacked authority to enforce smoking bans.
**Sole responsibility**	Participants felt solely responsible for advocating and enforcing smoke-free environments.
**Variable level of support for SHS bans**	Emotional support during hospitalization did not translate into sustained smoking behavior change.

Themes were derived from thematic analysis of interviews with 20 mothers of medically vulnerable infants.

### Household structure and power dynamics

Participants conveyed that household composition and relative power for decision making in the household impacted their ability to protect their infant from SHSe via the establishment of smoking bans in the home and car. Eight participants lived in multigenerational homes with their parents and/or other extended family members while twelve lived in a single generational home, two of which lived alone while the other ten lived with a romantic partner.

*Power dynamics in multigenerational homes*

The primary contributing factor to differences between these household structures is power differentials between the mother and the primary authority figure in the household. When one family member holds significant authority or influence, it can be challenging for others to enforce rules around smoking, especially if this person is resistant to change. This power differential can lead to conflicts and hinder efforts to maintain a smoke-free environment. In multigeneration homes, the primary authority figure – often the parent of the participant – was perceived to have more power to establish household smoking bans. Moreover, household smoking bans varied by smoking status of the primary authority figure who was responsible for setting and maintaining them. For example, if the primary authority figure smoked, then smoking was allowed within the household with limited input from other household members. Similarly, if the primary authority figure was a non-smoker, smoking was disallowed within the household without regard to the opinions of other household members. Primary authority household members established household smoking rules with minimal consideration of mothers’ wishes, and exhibited limited willingness to engage in ban adaptation, even with the introduction of a medically vulnerable infant into the household. Participants indicated limited agency to influence household smoking bans, and reported feeling angry and dejected about being unable to protect their infants from SHSe.

We use pseudonyms to report participants views. For example, one struggled to enforce smoking rules due to resistance from her mother, who continues to smoke indoors:

*‘A lot of times I just had to go with what [my parents] say. I had to just say, “Okay, this is not what I want or what I agree with, but this is what is going to go down”.’* (Kayla)

Similarly, another stated:

*‘My mom prefers for us not to say anything [about smoking], we keep our mouths shut when we don’t want to. We are never allowed to make any of the rules, even about smoking, they are always in charge. I don’t want to fight so I don’t say anything.’* (Martina)

The lack of collaborative decision-making makes it challenging for participants within multigenerational households to maintain a smoke-free environment. Participants who lived in multigenerational homes often shared a strong desire for independence and the ability to set their own household smoking rules. One who lives with extended family members, was asked how smoking was going since hospital discharge and she stated:

*‘It’s pretty good but you get tired at some point – you want to go and be by yourself. Sometimes you want your own place, to just set your own rules.’* (Laura)

Similarly, others shared that the lack of agency when living with extended family members impacted their ability to set and enforce smoking rules:

*‘My brother was smoking on the front over there [indicating in the home] and he [partner] got mad about it* … *he [partner] isn’t allowed to stay here. I have to follow their [parents’] rules since I live here. That’s why I want to move out. The smoking yea is intense, we don’t agree about it and other issues.’* (Anna)

*‘My mom actually gets upset about it [attempt to set smoking rules], says that like I could go and move out – I was hoping that by the time the baby came home [from the hospital], we would actually be out of here.’* (Belinda)

Dominance of the primary authority figure over home smoking policies may also be protective, however. For instance, Tasha lives within a household where the primary authority figure, the participant’s father, was a non-smoker and heavily opposed smoking. As such, a sibling who smoked did so only outside of the home or avoided smoking altogether, effectively reducing infant SHSe.

*Power dynamics in single generation homes*

The lack of agency and power experienced by participants in multigenerational homes was less often reported when participants lived with their partner or alone, referred to here as single-generational homes. They reported an increased ability to set and enforce household smoking bans. Maria and her partner worked together to reduce smoking, especially after their baby’s hospital experience. They used visual reminders and discussed the impact of smoking on their baby’s health, reinforcing their shared goal of creating a healthier environment.

Further, when household smoking rules were broken, participants felt more power to engage in conversations with their partners to reinforce smoking rules. Sarah’s partner initially started smoking outside the home but slowly started resuming smoking in the home, although in a different room:

*‘One of the main things to keep our relationship ok is having a good flow of communication, which I can’t say we always do, but I know that’s a good way to solve our problems* … *it has in the past when he started smoking again, that’s one of the main things to think of, how can I communicate to him how I’m feeling or how I feel. And by me opening up it does allow him to understand and I listen, too. He was like, yeah, we got to do it for the baby.’* (Sarah)

However, even within single generational homes, mothers reported feeling primarily responsible to protect their infant from SHSe.

### Sole responsibility

Mothers reported feeling that they had the sole responsibility to protect their infants from SHSe. In our intervention, partners and family members were invited to participate in the counseling sessions; however, the majority of visits were attended solely by mothers. Mothers reviewed educational materials during the sessions that could be shared with other household members. Participants reported actively attempting to influence the behavior of individuals who smoke within their household by asking them to stop smoking, or smoke outside, and/or switch to electronic cigarettes (which some felt was a safer option). Women described being the primary person responsible for implementing SHSe rules and reported a lack of support from other household members, which hindered their ability to establish a smoke-free environment. They felt the onus of enforcing and maintaining SHSe rules was on them and these exchanges often resulted in conflicts and minimal changes.

One shared that she felt pressure to advocate for her children to protect them from her partner’s smoke exposure:

*‘He knows how I feel about it, he knows how I protect from it [smoking] for my own health and then with the kids because they can’t defend themselves. I’m their advocate, also. So it’s out there. And he knows.’* (Melissa)

Another shared something similar:

*‘He [partner] would look at them [intervention materials] and, and I would tell him, John, you know, wash your hands. So, I noticed he started to smoke a cigarette, go home, and be around the baby. And I told him, ‘nicotine is in your shirt and your skin. So, when you smoke you got to wash your hands or you change your shirt, or wear a jacket, and leave it outside.’* (Judy)

One felt it was her responsibility to educate her partner on the strategies she was learning to mitigate SHSe:

*‘…He knew that it [smoking] bothered me and then eventually when he continues to* … *I would let him know, “Okay, we talked about smoking before. I told you everything I learned [from the study], I thought you stopped. I didn’t know that this was something you were going to still do.” It caused a lot of arguments between us. He felt like I was always bossing him around.’* (Valentina)

Another shared:

*‘My husband is very stubborn. I would ask him not to smoke in the car, and he’d be like, “Well it’s not going to kill him.” And I’m like, “But you have to understand, smoking is bad, I don’t like the smoking* … *it bothers me.” He just tells me “Oh, it’s going to be okay” and I, I cannot get him to get to that point where he don’t smoke in the car. We fight about it all the time.’* (Ramona)

Many household members who initially made changes to their smoking eventually returned to their previous smoking habits:

*‘I loved it [the study], especially in the beginning. It changed a lot inside the home* … *in the beginning she [participants’ mother] wouldn’t smoke in the house at all, because I guess the information that I was bringing back to her she didn’t even know. So she was real concerned about not getting the baby sick because she was in the hospital for so long after she was born, you don’t want to make it worse by, you know, smoking. So it was, it was good before, but it’s, now it, everything’s just went back to normal.’* (Jessie)

*‘At first, when I told her what I learned [from the study] she would only smoke outside. And then it went to she’ll just smoke in the restroom and we’ll be in a different room while she smokes. I think at first, she, she was a little scared about it. But then she goes right back to the same things* … *she just gave up on trying to change’* (Alice)

### Variable level of support for SHS bans

Familial and partner support to manage the stress of having a medically vulnerable infant was reportedly high for most participants; however, this support often did not translate to smoke-reduction efforts post-infant discharge. Participants described the importance of a strong familial support system where family members collaboratively contributed to aid each other during the difficult time of having an infant in the hospital. However, familial support varied by levels of cohesiveness. For those participants with strong, close-knit relationships, support was perceived as essential and important to manage difficult circumstances.

One shared that her family is very close and she relies heavily on them as a source of emotional support during her infants’ stay in the hospital:

*‘I would visit and I had a couple people in my family to talk to that I could trust. I went to my parents’ house a lot to get breaks.’* (Maria)

Similarly, another shared that her family members helped her cope and lifted her spirits when she was feeling discouraged about her baby’s health:

*‘When I get down they always help me see the good side of everything. They’ll say “Look, he’s [baby] still healthy in every way he possibly can be. He’s still happy. He still does normal stuff, everything about him exceeds a normal five-month-old”* … *they remind me that his adjusted age is only three-and-a-half months.’* (Raquel)

In addition to emotional support, participants shared how extended family members also provided financial support:

*‘My family is the only ones that help me – especially my dad. He’s the one who got me the apartment and paid for it until now that we’re starting back to work, now that everything’s okay with Emma.’* (Regina)

Within families with less family cohesiveness, the influence by extended family members was perceived as non-supportive and contributed to conflicts within the household. For instance, one shared that tension within the family arose when her mother-in-law attempted to exert authority and influence childrearing decisions that differed from those of the participant:

*‘We did have some issues with his mom, because, she kind of wanted things her way, and that’s when we used to actually [sleep] overnight with the baby in the hospital, so she [partners’ mom] would stay with me, but to be honest with you, I thought that only made things more difficult.’* (Martina)

Similar to familial support, receiving support from romantic partners was a major contributor to managing the stressful experience of having a medically fragile infant in the hospital. Participants reported how the level of support they received impacted their primary relationships. For one, having the support of her partner during her babies’ hospital stay positively impacted the cohesiveness in her primary relationship:

*‘Before we had her we [her partner and her] would fight over little dumb things or argue, but now with everything that happened [having a baby in the hospital] and how it happened, and he was there through the whole thing, I mean, I don’t know, we’re just now more close together, and our only concern is the baby and making her happy and having a good, strong family.’* (Valentina)

Participants shared that support was an important contributor to help participants feel understood, valued, and cared for while their babies were in the hospital, but this support did not generalize to implementing smoking household rules. For instance, one shared that her mother and sister were highly supportive of her while she recovered from her cesarean section and her infant was in the hospital. She went on to share that these same family members were not willing to make changes pertaining to secondhand smoke reductions in the home:

*‘When it comes to smoking, we don’t, we don’t always agree with each other. It’s actually why I want to move out.’* (Beatrice)

Similarly, another who reported receiving a great deal of familial support during her hospital stay shared the following about her mothers’ continued smoking:

*‘So, she was real concerned about not getting her [baby] sick because she was in the hospital for so long after she was born, you don’t want to make it worse by, you know, smoking. So, it was, it was good before, but everything’s just went back to normal. They don’t help support me with it at all.’* (Valeria)

## DISCUSSION

This study provides critical insights into the complex social and familial dynamics that influence the implementation and maintenance of home smoking bans among families with medically vulnerable infants. Despite the well-documented risks of SHSe, particularly for medically fragile infants, our findings underscore the persistent challenges caregivers face in creating smoke-free environments. These challenges are highly influenced by household composition and power dynamics, as well as the perception of caregiving responsibilities.

The themes we identified support the Behavioral Ecological Model (BEM)^9^, which emphasizes the role of environmental and social contingencies in shaping health behaviors. Mothers in multigenerational households often lacked the authority to enforce smoking bans, especially when the primary authority figure was a smoker. This aligns with previous research indicating that women’s ability to control SHSe is often constrained by gender norms and household hierarchies^19,28^. Conversely, mothers in single-generation households reported greater agency, though they still faced challenges in negotiating smoking behaviors with partners.

Currently, no efficacious interventions have been identified that reduce SHSe in infants. Motivational interviewing (MI) has shown to have promising effects on targeting smoking cessation, and multiple studies have tested its utility to reduce SHSe with limited success. Walker et al.^29^ employed a home-based intervention using MI to reduce SHSe among children. The intervention included home visits by trained counselors who used MI techniques to engage primary caregivers in discussions about smoking behaviors and strategies to reduce SHSe. However, while the study results found that MI was effective in increasing caregiver awareness and promoting protective behaviors, such as smoking outside and establishing home smoking bans, it did not result in significant reductions in child SHSe biomarkers. The lack of MI intervention success on SHS reductions may result from the failure to address contextual household factors that impact SHSe strategy adoption among household members. It is clear from our results that household members are supporting new mothers in various ways. However, this support did not consistently extend to smoking-related behavior change. This disconnection suggests that while household members may rally around the mother and infant during an acute medical crisis, they may not perceive SHSe as an urgent or shared responsibility. This gap presents a critical opportunity for intervention: specifically leveraging existing support systems into SHSe interventions to increase motivation and extend their influence on health-protective behaviors post-discharge. Importantly, interventions on SHSe delivered solely to primary caregivers are unlikely to be effective. The theme of sole responsibility highlights the disproportionate burden placed on mothers to advocate for and enforce SHSe rules. Despite being the primary caregivers, they often lacked support from other household members, leading to conflict and emotional strain, especially when household members are not ready to change. This finding is consistent with prior studies showing that interventions targeting only mothers may be insufficient, particularly when they are not the primary smokers or lack decision-making power in the household^30-32^. Without adaptations for contextual household circumstances, SHSe intervention may be inadequate to effectively protect infants from SHSe, especially when individuals live within multi- or single-generational homes.

Changes in our approach to SHSe interventions are needed. Specifically, SHSe interventions must move beyond individual-level behavior change and address the broader household environment and, importantly, should include household smokers who may not be the primary caregivers. This change may alleviate the dissemination burden on new mothers who are contending with the stress of having a medically fragile infant in the home. A study on group-based MI, albeit for smoking cessation, found that the treatment was effective in increasing quit attempts and reducing cigarette consumption compared to standard care. MI group sessions focused on fostering support, enhancing motivation, and building commitment to quit smoking. The group format allowed participants to share experiences, reinforce each other’s goals, and develop collective strategies for overcoming barriers^33^.

A shift in delivering a family group intervention to all household members has the potential to minimize household conflict, harness support, and increase the collaborative nature of individuals residing in households with multiple individuals. However, these Collaborative Care Models must be contextually grounded and inclusive of the household’s social dynamics. These models should ideally engage all household members, but at a minimum, they must include the infant’s primary caregiver and, critically, any resident smoker(s). This inclusive approach ensures that the responsibility for maintaining a smoke-free environment does not fall solely on the mother, who often lacks the authority or support to enforce such rules alone^32^. Additionally, increasing motivation for smoking cessation and offering evidence-based pharmacological treatments, such as nicotine replacement therapy (NRT), medications (e.g. varenicline), and other cessation aids, are important strategies targeted to household smokers in order to reduce SHSe effectively^34^. When combined with behavioral counseling, pharmacological aids can double the chances of successful cessation, offering a powerful complement to household-based interventions aimed at reducing SHSe^35^.

Further, it may be possible to leverage technology and wearable devices – such as air quality monitors that detect indoor particulate matter from tobacco smoke or mobile apps that provide feedback on SHSe levels and track smoking triggers. Providing motivational messaging based on this feedback can further enhance awareness and accountability among household members^36,37^. These smart technology tools offer personalized feedback, progress tracking, and coping strategies, reinforcing behavior change through real-time environmental monitoring to reduce SHSe in homes with children^38^. A study by Klepeis et al.^39^ provided families with monitors that measured indoor particulate matter in the air associated with tobacco smoke. The devices offered real-time, objective feedback about air quality, alerting household members when smoke levels were elevated. This immediate feedback mechanism helped reinforce smoking bans by making the invisible harms of secondhand smoke visible, thereby increasing awareness and promoting behavior change among smokers in the home. Moreover, the immediate feedback by the external monitor alleviated the baby’s primary caretaker, often a non-smoker, from bearing the primary responsibility of asking household members who smoked to not smoke.

### Limitations

While this study provides rich insights into participants’ experiences with setting and maintaining smoking bans within their homes, several limitations should be noted. First, the sample size was relatively small and drawn from a single geographical region, which may limit the transferability of findings to other populations. Second, participants were selected from a larger clinical trial drawn from a NICU population, potentially introducing selection bias to those individuals willing to participate in research, who may have strong opinions or particular experiences that differ from those uninterested in research study participation. Third, the data were collected via semi-structured interviews, which rely heavily on participants’ ability to articulate their thoughts and on the researcher’s interpretive lens. As such, researcher bias may have influenced the thematic analysis despite efforts to maintain reflexivity and rigor. Finally, the study did not include longitudinal follow-up, so it captures only a snapshot in time rather than evolving perspectives.

## CONCLUSIONS

Integrating tools such as NRT, medications, and technology into a family-centered Collaborative Care Model may enhance intervention reach and impact. By embedding these interventions and considering household contextual circumstances we can better support families in maintaining cohesiveness and support while collaboratively working to protect their household infant from risks associated with secondhand smoke exposure.

## Data Availability

The data supporting this research are available from the authors on reasonable request.
